# Parental support in promoting children’s health behaviours and preventing overweight and obesity – a long-term follow-up of the cluster-randomised healthy school start study II trial

**DOI:** 10.1186/s12887-019-1467-x

**Published:** 2019-04-11

**Authors:** Åsa Norman, Zangin Zeebari, Gisela Nyberg, Liselotte Schäfer Elinder

**Affiliations:** 1 Department of Public Health Sciences, Karolinska Institutet, 171 77 Stockholm, Sweden; 2 Jönköping International Business School, Gjuterigatan 5, Box 1026, 551 11 Jönköping, Sweden; 3 The Swedish School of Sport and Health Sciences, Lidingövägen 1, 114 33 Stockholm, Sweden; 4 Centre for Epidemiology and Community Medicine, Stockholm County Council, Box 1497, 171 29 Solna, Sweden

**Keywords:** BMI-sds, Diet, Intervention, Motivational interviewing, Physical activity, Quantile regression, School, Screen time, Sedentary behaviour, Socio-economic position

## Abstract

**Background:**

Effects of obesity prevention interventions in early childhood are only meaningful if they are sustained over time, but long-term follow-up studies are rare. The school-based cluster-randomised Healthy School Start (HSS) trial aimed at child health promotion and obesity prevention through parental support was carried out in 31 pre-school classes (378 families) in disadvantaged areas in Sweden during 2012–2013. Post-intervention results showed intervention effects on intake of unhealthy foods and drinks, and lower BMI-sds in children with obesity at baseline. This study aimed to evaluate the long-term effectiveness 4 years post-intervention.

**Methods:**

Data were collected from 215 children in March–June 2017. Child dietary intake, screen time, and physical activity were measured through parental-proxy questionnaires. Child height and weight were measured by the research group. Group effects were examined using Poisson, linear, logistic, and quantile regression for data on different levels. Analyses were done by intention to treat, per protocol, and sensitivity analyses using multiple imputation.

**Results:**

No between-group effects on dietary intake, screen time, physical activity, or BMI-sds were found for the entire group at the four-year follow-up. In girls, a significant subgroup-effect was found favouring intervention compared to controls with a lower intake of unhealthy foods, but this was not sustained in the sensitivity analysis. In boys, a significant sub-group effect was found where the boys in the intervention group beyond the 95th percentile had significantly higher BMI-sds compared to boys in the control group. This effect was sustained in the sensitivity analysis. Analyses per protocol showed significant intervention effects regarding a lower intake of unhealthy foods and drinks in the children with a high intervention dose compared to controls.

**Conclusions:**

Four years after the intervention, only sub-group effects were found, and it is unlikely that the HSS intervention had clinically meaningful effects on the children. These results suggest that school-based prevention programmes need to be extended for greater long-term effectiveness by e.g. integration into school routine practice. In addition, results showed that children with a high intervention dose had better long-term outcomes compared to controls, which emphasises the need for further work to increase family engagement in interventions.

**Trial registration:**

ISRCTN, ISRCTN39690370, retrospectively registered March 1, 2013, http://www.isrctn.com/ISRCTN39690370.

## Introduction

Overweight and obesity comprise serious threats to health, causing increased morbidity and mortality globally [1]. In Sweden, a strong socioeconomic gradient in obesity is seen among both adults [2] and children [3, 4]. Obesity tracks to some extent from childhood to adolescence and adulthood [5], which points to the importance of prevention early in life through the promotion of healthy dietary habits and physical activity, and a reduction in sedentary behaviour. Research has shown that parents constitute an important target group for obesity prevention interventions in younger children. Therefore, parental involvement has been strongly emphasised in interventions to promote health and prevent unhealthy weight development in children [6, 7]. Based on this, the Healthy School Start (HSS) intervention was developed in Sweden in 2010 [8] with the aim through school-based parental support of promoting healthy behaviours and preventing unhealthy weight development among children. The intervention was specifically developed for children starting school (5 to 7 years old) in disadvantaged areas and included a follow-up measurement 5 months post-intervention. The HSS intervention was evaluated in two cluster-randomised trials, in 2010–2011 with 243 children in families with low to middle socioeconomic position (SEP), and in 2012–2013 with 378 children in families with low SEP. The results of the first trial showed significantly higher vegetable intake in the intervention group compared to the control group post-intervention, and higher total physical activity among girls at weekends [9]. The effect on vegetable intake was sustained for boys at the five-month follow-up [9]. Post-intervention results from the second trial showed a significantly lower intake of unhealthy foods and drinks in the intervention group compared to controls, and a decrease in BMI-sds in children who were obese at baseline [10]. The effect on unhealthy foods was sustained in boys in the intervention group at the five-month follow-up.

Important public health gains from health promotion and prevention interventions, such as an increase in the proportion of individuals with normal weight, take time to develop, and it is therefore recommended to do long-term follow-up of trials [11, 12]. Delayed effects have been seen after 1 to 2 years in some child obesity prevention interventions [13, 14]. Unfortunately, long-term follow-up studies including a time period of more than 1 year are scarce for reasons such as the wait-list control groups being offered the intervention after the trial, the limited funding of trials, and/or difficulties in locating participants after several years.

This study aims to evaluate the long-term effectiveness after 4 years of the Healthy School Start II intervention, a parental support programme to promote health and prevent obesity in children in the school setting.

## Methods

The HSS II intervention was carried out during 2012–2013 in three disadvantaged areas in Stockholm County with a high prevalence of overweight and obesity among children in the county [15]. The intervention was evaluated as a parallel group cluster-randomised controlled wait-list trial in pre-school classes (five- to seven-year-old children) with school class as the unit of randomisation [10]. The control group was offered the intervention after the five-month follow-up measurements. Thirteen schools with 31 pre-school classes participated at baseline with a total of 378 children. Outcome measurements regarding children’s diet, physical activity, screen time, height, and weight were taken at baseline in August and September 2012 (T1), post-intervention in April and May 2013 (T2), at a five-month follow-up in September and October 2013 (T3) [10], and during March to June in 2017 (T4) for this four-year follow-up study.

The Healthy School Start intervention.

The HSS is based on Social Cognitive Theory [16] with a published study protocol [8] and includes three intervention components:

### Health information to parents

A brochure developed specifically for the intervention containing evidence-based advice regarding healthy dietary, physical activity, screen, and sleeping habits for six-year-old children. The brochure is written in basic, easy-to-read Swedish and also available in Arabic and Somali, which were common languages in the intervention areas. As a booster to the information in the brochure, an information group meeting was offered in each of the intervention schools.

### Motivational interviewing (MI) with parents

One to two sessions of MI per family were offered, where parents had the opportunity to focus on a target behaviour regarding their child’s diet or physical activity in the home environment that they wanted to change. Two counsellors, with documented MI competence prior to the intervention, conducted the MI sessions.

### Classroom activities with home assignments

Ten 30-min sessions were conducted by the teachers with support from a programme-specific teachers’ manual and tool-box. Classroom sessions were complemented by home assignments to be completed by the child and parents together in a workbook.

Fidelity to the intervention components was monitored during implementation [10].

### Participation in intervention by the control group

In line with the wait-list design, control classes were offered to take part in the intervention components after the five-month follow-up measurements were completed in October 2013 as follows: Component 1: The brochure was sent home to all parents in the control group who had consented to participate in the trial (*n* = 193), but the parents of only one child (less than 1% of the control group) participated in the information meeting offered. As sending home information in itself has a very limited effect on behavioural change [17, 18], this was not seen as an obstacle to a long-term follow-up.

Component 2: Only two (1%) of the 193 control parents chose to participate in the MI session.

Component 3: All teachers in the 15 control classes were offered the classroom material and workbooks to be used in class from November 2013 until May 2014. Three of the 15 classes did not conduct any of the lessons; five classes gave two of the lessons, two classes gave six lessons, and two classes gave all ten lessons. Teachers in three of the 15 classes did not respond to the queries about whether the material had been used or not.

### Data collection

All 378 families from the baseline measurements were targeted for inclusion in the four-year measurement (T4). Contact with the families was re-established through several steps. First, schools were contacted and reminded about the planned data collection and asked to provide contact details for parents in the families included. In some cases, we had difficulties in establishing contact with schools due to staff turn-over, including school principals, which also made it difficult to get into contact with parents. Classes had been reorganised and children had changed school class. In addition, two of the schools had merged into one and several children had moved to schools not included in the HSS II study.

### Children’s health behaviours

Children’s diet, physical activity, and screen time were measured by means of a parent report consistent with the previous assessments [10] using the Eating and Physical Activity Questionnaire (EPAQ) [19]. Regarding diet, parents responded to their child’s dietary intake during the previous weekday. Items included fruits and vegetables, snacks, sweets/chocolate, ice-cream, cakes/buns/cookies, soft drink, flavoured milk and fruit juice in order to capture indicators corresponding to healthy and unhealthy dietary intake, respectively. The response scale included whole servings in the categories: 0, 1, 2, 3, 4, or 5 or more servings for food items, and 0, 1, 2, 3, 4, 5, 6 or more servings for drink items. Servings were defined as: drinks = 1.5 dl, vegetables = e.g. 2 dl grated carrots/cabbage or a large tomato or 2–3 broccoli heads, fruit = e.g. a small apple or about 10 grapes, snacks = 1.5 dl crisps or cheese doodles, sweets = about 1.5 dl of sweets or 4 pieces from a chocolate bar, cakes = a small bun or 5 small biscuits, ice-cream = a small ice cream bar or 1 dl of ice-cream. Aggregated dietary indicator variables were created as the sum of either healthy foods (fruit and vegetables), unhealthy foods (snacks, sweets/chocolate, ice-cream, cakes/buns/cookies), or unhealthy drinks (soft drink, flavoured milk and fruit juice above one serving). Dietary items of EPAQ have been validated against 24-h recall in an Australian context with significant correlations between the two methods for different items ranging from r = 0.57 to r = 0.88 [19].

In addition, the questionnaire measured whether the child was active in organised activity, i.e. a member of, and active participant in an organisation delivering organised activity such as swimming, basketball, or capoeira, for children, (yes or no), and minutes of screen time in front of the television or computer during the previous weekday. The questionnaire was available in Swedish and distributed via a web-link.

### Children’s anthropometry

Height and weight were measured in school according to standardised procedures [8] by two trained research assistants. The standardised procedure included measuring the child’s weight where the child was wearing light clothing (t-shirt and trousers) to the nearest 0.1 kg (kg) using a digital scale (SECA Robusta 813).Height was measured using a SECA stadiometer (214) to the nearest 0.001 m (m). The child was instructed to take off shoes, stand with the feet apart, having the calves, back and shoulders touching the stadiometer, and the heels and back touching the wall and looking straight forward. The research assistants were trained in the measurement procedures to the level of reliability where they differed 0.1 kg in the weight measurement and 0.002 m in the height measurements, when measuring the same person, before they started the T4 measurements in this study. The assistants measured both intervention and control group to an equal extent. BMI was calculated as weight (kg) divided by height (m) squared, and BMI standard deviation score (BMI-sds) was calculated according to a Swedish reference standard [20]. The International Obesity Task Force cut-off points were used to define children’s weight status (underweight, normal weight, overweight, and obesity) [21].

### Socio-economic position

Parental educational level and area of residence were used as indicators of SEP [22, 23]. The study setting comprised three areas in Stockholm County with low employment and low educational level that were specifically targeted by the government in order to increase socio-economic development [24]. Furthermore, the highest self-reported educational level attained by either parent in the family at T1 was used as an indicator of SEP. The SEP variable was dichotomised: low education as equal to primary and secondary school (≤12 years of schooling) and high education (> 12 years of schooling) equal to third level education.

### Region of birth

Parents reported their country of birth at T1. The family was categorised as originating from outside the Nordic region (Sweden, Norway, Finland, Denmark, and Iceland) if one or both parents were born outside the region.

### Statistical analyses

**Fig. 1:**
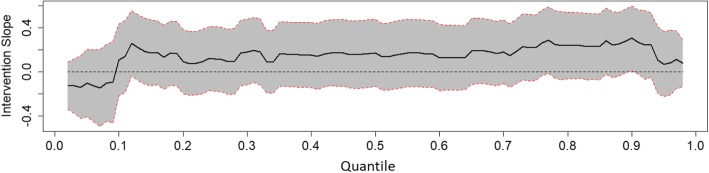
Effect of intervention on BMI-sds of the intervention group relative to the control group along the 2th up to the 98th percentiles. Results of Quantile regression of BMI-sds with adjustment for baseline value, sex of child, and parental education (intention to treat). Subjects are dependent observations between T1 and T4 with valid measurements at T2. Line represents quantile regression coefficient estimates of intervention group (with the control group as reference). Grey area represents 95% confidence intervals

**Fig. 2:**
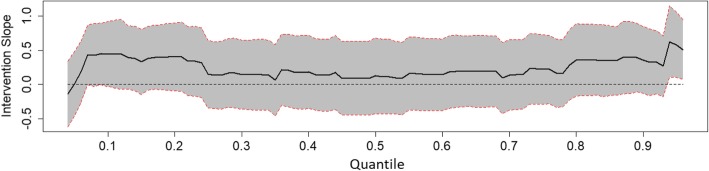
Effect of intervention on BMI-sds of the intervention group relative to the control group along the 4th up to the 96th percentiles, boys. Results of Quantile regression of BMI-sds with adjustment for baseline value, sex of child, and parental education (intention to treat). Subjects are dependent observations between T1 and T4 with valid measurements at T2. Line represents quantile regression coefficient estimates of intervention group (with the control group as reference). Grey area represents 95% confidence intervals

**Fig. 3:**
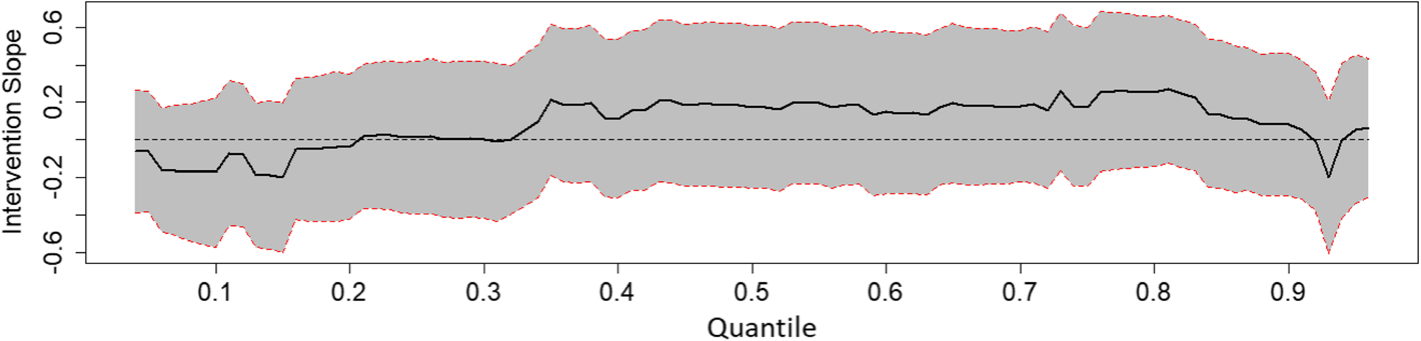
Effect of intervention on BMI-sds of the intervention group relative to the control group along the 4th up to the 96th percentiles, girls. Results of Quantile regression of BMI-sds with adjustment for baseline value, sex of child, and parental education (intention to treat). Subjects are dependent observations between T1 and T4 with valid measurements at T2. Line represents quantile regression coefficient estimates of intervention group (with the control group as reference). Grey area represents 95% confidence intervals

Baseline differences between intervention and control group of individuals who were included in measurements at T4 were examined using an independent sample t-test for continuous data and Chi-square for categorical data. Long-term effectiveness of the intervention was evaluated using the same procedure as in the previous study [10]. Thus, values at T4 were compared to values at T1. Only individuals with valid values at T2 were included in the analyses in order to obtain a sample comparable to our previous effectiveness evaluation post-intervention. Analyses of long-term effectiveness were undertaken in several steps, as has been recommended by Little et al. [25]. The analyses were performed as follows:

1. Complete cases intention to treat (ITT) analysis was performed with individuals that had valid data at T1, T2 and T4 (*n* = 215) regardless of their degree of participation in the intervention activities. This analysis represents the main analysis and is presented in Tables 2, and 3, and Figs. 1, 2, and 3.
2. A per protocol analysis, which included children from families who had participated in both MI sessions, as the MI sessions were hypothesized as being the main intervention component. In total, this analysis included 88 to103 families depending on the outcome.
3. A multilevel analysis with two levels (individual and school class) was performed in order to adjust for between-school class differences (school class constituting the original unit of randomization, *n* = 31). In these analyses, a random intercept for school class clustering was estimated using the maximum likelihood estimation method. A likelihood ratio test was used to compare model fit between the models with and without the random intercept.
4. A sensitivity analysis was undertaken for significant outcomes (unhealthy foods, and BMI-sds) in order to detect whether effects were sustained when missing data was accounted for. For the sensitivity analysis multiple imputation was performed using five imputed datasets including all available variables regarding demographics, diet, activity, and anthropometry to include the total sample at T1 (*n* = 378). As the missing data had a random pattern, the fully conditional specification method was used to generate imputed data [26].

To determine long-term intervention effects a crude model was first tested for all outcomes at T4 using the group as the predictor with adjustment for baseline values. Second, the main model including group, sex of the child, parental education, and baseline values, was tested. Third, interactions between group and sex, or group and parental education were tested. Analyses were stratified if significant interaction terms were found. For the continuous outcome (screen time), linear regression was performed. For count outcomes (single and aggregated food, and drink variables), Poisson regression was performed. For the binary outcome (child active in organised activity yes/no), logistic regression was performed. To analyse the effect of the intervention on a wide spectrum of BMI-sds, quantile regression was applied. The conditional quantiles of the BMI-sds at T4 (conditioned on the BMI-sds at T1) were modelled for a wide range of percentiles (as far as the estimable percentiles below the 5th and above the 95th percentiles).

In addition to the regression analyses, differences in changes between the intervention and control group regarding the prevalence of weight status (underweight, normal weight, overweight, and obesity) between T1 and T4 were examined using a difference in difference approach and tested for statistical significance using independent samples t-test.

All analyses were performed using the SPSS 23.0 software package (Chicago, Illinois, USA), except for the multilevel analysis where MLwiN (version 2.36, 2014, Bristol University, UK) was used, and the quantile regression analysis where quantreg library of the statistical package R was used [27]. The level of significance was set to *p* < 0.05.

## Results

The following number of children were included in each measurement: Baseline (T1) *n* = 378, post-intervention (T2) *n* = 359, five-month follow-up (T3) *n* = 345, and four-year follow-up (T4) *n* = 215. Of the 163 children (intervention *n* = 88, control *n* = 75) that were lost to follow-up at T4, 20 had moved, 19 declined participation, 11 were not present at the time of anthropometric measurement, and 113 could not be contacted or it was not possible to book anthropometric measurements for them. No statistically significant differences were found regarding characteristics of participants included at T4 (n = 215) and the total sample at (n = 378) at baseline (not shown). Characteristics at baseline for participants measured at T4 are displayed in Table 1, including the number of respondents for each variable. No significant differences were found between the intervention and the control group at T1, but the control group had a higher intake of unhealthy foods (*p* = 0.05).

**Table 1:** Descriptive characteristics at baseline (T1) categorised by intervention and control group

	Total	Intervention	Control	*p*	*n*
	*n* = 215	*n* = 97	*n* = 118		
	Mean (SD)	Mean (SD)	Mean (SD)		
Girls (%)	49.3	53.6	45.8	0.25	215
Age (years)	6.3 (0.3)	6.3 (0.3)	6.3 (0.3)	0.93	215
Parental low education per family (%)	51.2	50.0	52.3	0.74	203
Parents born outside the Nordic region (%)	87.9	85.3	90.2	0.3	207
Anthropometry					
Waist circumference (cm)	56.7 (5.9)	56.7 (6.2)	56.6 (5.1)	0.91	215
Body mass index (kg/m2)	16.9 (2.5)	17.0 (2.6)	16.9 (2.5)	0.75	215
BMI sds^a^	0.71 (1.41)	0.75 (1.39)	0.67 (1.43)	0.71	215
Normal weight^b^ (%)	67.0	69.1	65.3	0.56	215
Overweight^b^ (%)	15.3	15.5	15.3	0.97	215
Obese ^b^ (%)	11.6	11.3	11.9	0.90	215
Underweight^b^ (%)	6.0	4.1	7.6	0.28	215
Screen time					
Television/computer time (minutes/day)	129 (71)	127 (74)	130 (68)	0.75	178
Physical activity					
Children active in organised activity (%)	47.3	52.4	42.2	0.26	129
Diet (servings the previous day)					
Fruit juice^1^	0.61 (0.73)	0.62 (0.79)	0.6 (0.66)	0.73	148
Soft drink^1^	0.28 (0.54)	0.24 (0.49)	0.33 (0.58)	0.45	138
Flavoured milk^1^	0.30 (0.60)	0.21 (0.41)	0.39 (0.73)	0.21	139
Vegetables^1^	1.07 (0.80)	1.01 (0.82)	1.12 (0.77)	0.63	168
Fruits^1^	1.67 (1.0)	1.6 (0.88)	1.76 (1.08)	0.67	175
Snacks (crisps and cheese doodles)^1^	0.33 (0.66)	0.25 (0.52)	0.41 (0.77)	0.19	157
Chocolate/sweets^1^	0.53 (0.74)	0.42 (0.69)	0.64 (0.79)	0.17	165
Ice-cream^1^	0.52 (0.80)	0.35 (0.59)	0.69 (0.93)	0.08	168
Cake/buns/cookies^1^	0.55 (0.75)	0.48 (0.69)	0.62 (0.79)	0.57	164
Unhealthy foods^2^	1.77 (2.22)	1.37 (1.94)	2.16 (2.40)	0.05	173
Healthy foods^2^	2.90 (1.56)	2.87 (1.71)	2.92 (1.4)	0.7	177
Unhealthy drinks^2^	0.61 (1.08)	0.54 (1.15)	0.67 (1.01)	0.37	161

p = between intervention and control groups

BMI sds: body mass index standard deviation score,

^a^Defined according to Karlberg et al. [20]

^b^Defined according to Cole et al. [21]

^1^Serving sizes (examples below):

Snacks = 1.5 dl of crisps or cheese doodles

Sweets = about 1.5 dl of sweets or 4 pieces from a chocolate bar

Cakes = a small bun or 5 small biscuits

Ice-cream = a small ice cream bar or 1 dl ice-cream

Drinks = 1.5 dl

Vegetables = 2 dl grated carrots/cabbage or a large tomato or 2–3 broccoli heads

Fruits = a small apple or a bunch of grapes (about 10)

^2^Aggregated variables: unhealthy foods (snacks, sweets/chocolate, ice-cream, cakes/buns/cookies), healthy foods (fruit and vegetables) and unhealthy drinks (soft drink, flavoured milk, and fruit juice > 1 serving)

### Diet

The parental response rate to the dietary questionnaire at T4 ranged from 30 to 35% of the total sample at T1 for the different items.

Results of Poisson regression using the complete cases ITT approach showed a trend towards a healthier intake of foods and drinks favouring intervention on seven of the nine single food outcomes and on all aggregated food outcomes, but with no significant effect regarding the entire intervention group (Table 2). A significant sub-group effect regarding the intake of unhealthy foods was found for girls in the intervention group who had a lower intake (b = − 0.61, *p* = 0.03) at T4 compared to girls in the control group. In the sensitivity analysis using multiple imputation the effect remained in the same direction but was no longer significant. The multilevel analyses rendered results in the same direction as the ITT analyses. Analyses per protocol indicated a stronger, but non-significant, trend favouring intervention with larger regression coefficients and lower *p*-values regarding all food and drink outcomes. In the per protocol analysis, the intervention effect for girls regarding unhealthy foods reached statistical significance, as did an intervention effect on the entire group regarding intake of unhealthy drinks (*n* = 88, b = − 0.51, *p* = 0.04).

**Table 2:** Effects of intervention on dietary intake of indicator foods at T4 (intention to treat analysis)

Dietary intake - Servings^1^ the previous weekday^a^	n	b	p	95% CI	Unadjusted means (SD) at T4 per group			
					n	Int M (SD)	n	Cont M (SD)
Separate variables								
Snacks	109	−0.72	0.09	−1.55 to 0.12	52	0.15 (0.42)	57	0.40 (0.98)
Sweets/Chocolate	118	−0.25	0.41	−0.84 to 0.34	55	0.33 (0.51)	63	0.49 (0.91)
Cakes/Buns/Cookies	116	−0.53	0.07	−1.10 to 0.04	54	0.33 (0.67)	62	0.56 (0.86)
Ice-cream	124	0.03	0.96	−1.22 to 1.28	60	0.08 (0.42)	64	0.09 (0.34)
Soft drink/sugar syrup	87	−0.06	0.90	−0.87 to 0.76	42	0.26 (0.54)	46	0.30 (0.59)
Flavoured milk	84	−0.03	0.90	−1.02 to 0.95	42	0.19 (0.46)	42	0.21 (0.47)
Fruit juice	106	−0.31	0.17	−0.75 to 1.87	52	0.65 (0.84)	54	0.91 (1.17)
Vegetables	124	0.05	0.76	−0.27 to 0.37	60	1.25 (0.88)	64	1.19 (1.07)
Fruits	128	−0.23	0.14	−0.52 to 0.07	60	1.27 (1.13)	68	1.62 (1.21)
Aggregated variables^2^								
Unhealthy food					60	1.10 (2.12)	66	1.58 (2.0)
Girls^3^	**58**	**−0.61**	**0.03**	**−1.15 to − 0.61**				
Boys^3^	68	0.09	0.66	−0.31 to 0.49				
Unhealthy drink	114	−0.34	0.08	−0.71 to 0.04	56	0.84 (1.33)	58	1.24 (1.62)
Healthy food	133	0.11	0.30	−0.32 to 0.10	64	2.48 (1.59)	69	2.77 (1.90)
*Screen time*^*b*^	*n*	*b*	*p*	*95% CI*				
Television/computer time (minutes the previous weekday)	132	20.57	0.17	−8.63 to 49.77	63	148.79 (94.26)	70	136.16 (93.51)
*Physical activity* ^c^	*n*	*OR*	*p*	*95% CI*				
Child active in organised activity	127	1.77	0.16	0.79 to 3.95				

^a^Results of Poisson regression with adjustment for baseline, sex of child, and parental education (complete cases intention to treat)

^b^Results of Linear regression with adjustment for baseline, sex of child, and parental education (complete cases intention to treat)

^c^Results of Logistic regression with adjustment for baseline, sex of child, and parental education (complete cases intention to treat)

Subjects are dependent observations between T1 and T4 with valid measurements at T2

Bold - significant *p*-value < 0.05

b = regression coefficient, estimates of intervention group

OR = odds ratios for the intervention group

^1^Serving sizes (examples below):

Snacks = 1.5 dl of crisps or cheese doodles

Sweets = about 1.5 dl of sweets or 4 pieces from a chocolate bar

Cakes = a small bun or 5 small biscuits

Ice-cream = a small ice cream bar or 1 dl ice-cream

Drinks = 1.5 dl

Vegetables = 2 dl grated carrots/cabbage or a large tomato or 2–3 broccoli heads

Fruits = a small apple or a bunch of grapes (about 10)

^2^Aggregated variables: unhealthy foods (snacks, sweets/chocolate, ice-cream, cakes/buns/cookies), healthy foods (fruit and vegetables) and unhealthy drinks (soft drink, flavoured milk, and fruit juice > 1 serving)

^3^Stratified analysis due to interaction effect (group × sex)

### Physical activity and screen time

The parental response to the item measuring their child’s involvement in organised activity at T4 was 34%, and screen time 35% of the total sample at T1.

Results of linear regression using the complete cases ITT approach found no significant effects of intervention regarding minutes of screen time per weekday; nor did the logistic regression find any intervention effects on children’s involvement in organised activity (Table 2). The multilevel analyses and per protocol analyses rendered results in the same direction as the complete cases ITT analyses.

### Anthropometry

Height and weight were measured in 57% of the children at T4 of the sample at T1.

Results of the quantile regression on BMI-sds at T4 compared to T1 are shown in Figs. 1, 2, and 3. The graphs show the percentiles on the x-axis and the beta coefficient estimates for the intervention on the y-axis. A bold line represents the values of the beta coefficient estimates of the intervention across all the percentiles. Any point on the bold line above zero expresses a higher outcome (BMI-sds) for the intervention group compared to the control group at the corresponding percentile on the x-axis. The dotted lines are the 95% confidence intervals for the intervention coefficients. For a percentile, the intervention effect is significant only if the confidence interval at that percentile does not include the zero-line.

Figure 1 shows the intervention effect for BMI-sds along all quantiles where no significant effect is seen. A significant sub-group effect was found where boys in the intervention group had a higher BMI-sds around the last deciles compared to boys in the control group (Fig. 2). The effect remained significant and in the same direction in the sensitivity analysis using multiple imputation. No significant effect was seen among girls (Fig. 3). Analyses per protocol regarding the entire group rendered effects in the same direction, but somewhat stronger effects with generally greater regression coefficients.

Regarding the difference in prevalence of weight status (T1–T4), no significant difference was found between the intervention and control group (Table 3).

**Table 3:** Group difference in prevalence of weight status at T4

	T1				T4					
	Intervention (I^1^) *n* = 178		Control (C^1^) *n* = 181		Intervention (I^4^) *n* = 96		Control (C^4^)*n* = 113		Difference T1-T4	
Weight status^a^ (%)	*n*	*%*	*n*	*%*	*n*	*%*	*n*	*%*	*DD = (I* ^*4*^ *-I* ^*1*^ *)-(C* ^*4*^ *-C* ^*1*^ *)*	*p*
Underweight	11	6.2	11	6.1	6	6.3	6	5.3	0.9	0.99
Normal weight	121	67.7	122	67.4	52	54.2	72	63.7	−9,8	0.06
Overweight	30	16.9	25	13.8	26	27.1	24	21.2	2.8	0.71
Obesity	16	9.0	23	12.7	12	12.5	11	9.7	6.5	0.12

Results of independent samples t-test

*DD* difference in difference

p = between intervention and control groups

Subjects are dependent observations between T1 and T4 with valid measurements at T2

^a^Defined according to Cole et al. [21]

## Discussion

This long-term follow up of the HSS programme found no remaining significant intervention effects on dietary, physical activity, screen time outcomes or proportion of overweight and obesity 4 years after the intervention. However, a non-significant trend toward a healthier diet was found for the intervention group compared to the control, and a significantly lower intake of unhealthy food and unhealthy drink was found in the per protocol analyses. An unfavourable intervention effect was found regarding BMI-sds for boys over the 95th percentile, where boys in the intervention group had a significantly higher BMI-sds compared to boys above the same percentile in the control group. These results indicate that it is likely that the intervention had a minor influence on the participants after 4 years. The sub-group effect on boys previously found regarding a lower intake of unhealthy foods at the five-month follow-up [10] was not sustained after 4 years. Instead, at T4, a favourable sub-group effect was found for girls regarding a lower intake of unhealthy foods, which was not seen at T2 [10] and nor was it significant in the sensitivity analyses. However, in the per protocol analysis, the intervention group showed a significantly healthier dietary intake pattern, suggesting that the intervention had greater favourable effects in the children whose families had participated in the intervention to a greater extent. This finding indicates a positive dose-response relationship regarding the effects of the intervention. It underlines the importance of family engagement and compliance for health promotion and prevention interventions to be effective in the long term.

There are only a few health promotion or obesity prevention intervention studies with follow-up conducted as many as 4 years post-intervention with which we can compare our results. Regarding BMI, a four-year follow-up was conducted on the randomised controlled called AVall and was a school-based health education intervention targeting six-year-old children in Spain showed a significant BMI reduction with 1.13 kg/m2 for intervention children compared to controls [28]. The intervention lasted for 2 years and included health information such as healthy recipes for parents in addition to health education for children in school. The six-year-long controlled trial of the Cretan Health and Nutrition Education Programme, a school-based health education intervention in Greece, followed children from the first to the sixth grade [29]. Four years after the end of the intervention, a favourable intervention effect on BMI was found. In Germany, the school-based health educational intervention KOPS included five to seven-year-old children, lasted for 2 to 3 weeks and included an informational group-meeting for parents. The four-year follow-up study showed no intervention effect on BMI in the total sample. However, beneficial intervention effects were seen in the group with high SEP [30], possibly contributing to a greater socioeconomic gradient in overweight and obesity. A four-year follow-up was conducted on the 28-month EdAl school-based prevention intervention targeting adolescents (14–17 years) in Spain. The study found sub-group effects favouring intervention regarding a lower BMI z-score in girls and a lower prevalence of obesity in boys [31]. The intervention included a family component, but targeted an older age group compared to the HSS study. Regarding children in Sweden, only one long-term follow-up on a child obesity prevention intervention has been conducted to our knowledge. The Swedish PRIMROSE obesity prevention RCT included children at the age of 9 months and continued until the child was 4 years [32]. The intervention targeted parents, was conducted within the child health services, and lasted for 39 months. The follow-up was conducted 1 year after the end of the intervention at which time no effect on BMI or prevalence of overweight and obesity was found [32].

Even fewer long-term follow-up studies have included behavioural outcomes regarding physical activity, sedentary and dietary outcomes. Regarding diet, neither the EdA1, Cretan Health and Nutrition Education Programme or the KOPS study found any intervention effects after 4 years [29–31]. Regarding physical activity, the EdA1 study found significant intervention effects regarding hours per week in after school physical activity in boys, but the children were older than those in the HSS study. The Cretan Health and Nutrition Education Programme found a significantly higher moderate to vigorous activity in intervention group boys compared to boys in the control group [33], whereas no effects were found in the KOPS study [30].

Taken together, previous four-year follow-up studies of child health promotion and obesity prevention interventions mainly used health education targeting parents or children, and seldom included behavioural outcomes. Notably, all interventions showing effects 4 years after the end of intervention were conducted over several years [28, 29, 31, 33]. Systematic reviews of successful health promotion and obesity prevention interventions for younger children, regardless of long-term measurements, demonstrate active and extensive involvement by parents [6, 34] including face-to-face counselling [18], identification of barriers, self-monitoring, restructuring of the home environment, and goal-setting [34]. This is particularly true for families with low SEP [35] where the importance of prevention is greater compared to the general population. Furthermore, implementation studies have shown that successful adoption of interventions in clinics or institutions such as schools rely on the intervention being integrated into routine practice, and that the intervention activities facilitate the work of clinicians or teachers, who often experience a stressful and exacting work day. In addition, it is also important that the intervention can be adapted to the needs of providers and the target group [36–38]. The HSS intervention included face- to face counselling using MI where parents had the opportunity to identify barriers, the need for changes in the home, and setting goals in line with techniques found in other effective interventions [18, 34]. However, taken together, the three intervention components of the HSS intervention had a greater focus on knowledge about diet and activity, thus health education, than on healthy behaviours related to interaction and positive parenting around the food and physical activity in the family. A conclusion from a previous qualitative study on the target group found a need for increased focus on family interplay to possibly increase intervention effects [39]. Furthermore, the HSS intervention was limited to pre-school classes with an intervention period of only 5 months and the MI sessions were conducted by external counsellors, not by the school staff themselves. Based on extensive research, school-based parental support interventions are a promising route forward, but there is a need for programmes like the HSS to be extended over several years, and for family engagement to be increased, and to be fully integrated into the routine practice of school health care staff and teachers. Furthermore, future long-term follow-up studies of such interventions should include behaviour outcomes in addition to weight-related ones.

### Strengths and limitations

The use of quantile regression for analysing the BMI-sds comprises a strength of the study, since it allows for estimating differential effects for a wide spectrum of the BMI-sds scale rather than estimating the single point of the mean of BMI-sds, as is the case with least squares linear regression. In addition, quantile regression is more robust in the presence of outliers and problems with heteroscedasticity [40]. Furthermore, the inclusion of behavioural outcomes in addition to BMI constitutes a strength of the study, as this is rarely reported in long-term follow-up studies.

The main limitation of this study is the high attrition rate. We tried to compensate for this by performing various types of analyses including sensitivity analysis. The difficulty in retaining participants over long measurement periods comprises one of the greatest challenges to long-term follow-up [12]. However, 57% (*n* = 215) of the original participants, of whom the majority had a low parental educational level and whose parents were born outside the Nordic region, were retained, which is known to be a challenge [41, 42]. In the light of other long-term follow-up studies targeting families with low SEP, the retention rate was 59% in a one-year follow-up study on children in Israel [13], and 73% on a two-year follow-up study in children in the USA [14].

## Conclusion

Four years after the intervention, only sub-group effects were found, and it is unlikely that the five-month HSS intervention had clinically meaningful effects on the children 4 years after its completion. These results suggest that school-based health promotion and prevention programmes need to be extended in order to be effective long-term by e.g. integrating activities into school routine practice. In addition, results indicated that children of parents who had participated in the MI sessions had better long-term outcomes compared to controls, suggesting a dose-response relationship. This finding emphasises that further work to increase family engagement over time is also needed.

## Abbreviations

BMI-sds: Body Mass Index standard deviation score
HSS: Healthy School Start
ITT: Intention to treat
SEP: Socioeconomic position
